# Poverty in Lesbian, Gay, Bisexual, Transgender, Queer, Two-Spirit, and Other Sexual and Gender Minority (LGBTQ2S+) Communities in Canada: Implications for Social Work Practice

**DOI:** 10.1177/1049731521996814

**Published:** 2021-03-10

**Authors:** Hannah Kia, Margaret Robinson, Jenna MacKay, Lori E. Ross

**Affiliations:** 1 School of Social Work, The University of British Columbia, Vancouver, British Columbia, Canada; 2 Departments of English, Sociology, and Social Anthropology, Dalhousie University, Halifax, Nova Scotia, Canada; 3 Dalla Lana School of Public Health, University of Toronto, Ontario, Canada

**Keywords:** LGBTQ2S+ populations, poverty, intersectionality, relational poverty analysis, social work practice

## Abstract

In this article, we draw on a recent review of the Canadian literature on poverty in lesbian, gay, bisexual, transgender, queer, two-spirit, and other sexual and gender minority (LGBTQ2S+) communities to conceptualize social work interventions that may be used to address material inequities among these groups. Our literature review, which was based on a total of 39 works, revealed distinctive expressions of poverty among younger and older LGBTQ2S+ groups, as well as racialized, newcomer, and Indigenous sexual and gender minorities. Drawing on these insights, together with theoretical frameworks grounded in intersectionality and relational poverty analysis, we conceptualize these expressions of material inequity as salient sites of social work practice and propose interventions targeting these manifestations of LGBTQ2S+ poverty at various levels. Given the centrality of anti-poverty work as part of the social work profession’s commitment to social justice, and the dearth of social work literature on LGBTQ2S+ poverty, this article promises to make significant contributions to social work scholarship and professional practice.

The prominence of poverty in lesbian, gay, bisexual, transgender, queer, two-spirit, and other sexual and gender minority (LGBTQ2S+) populations is increasingly substantiated in a growing body of literature (Albelda et al., 2009; Kia et al., 2020; Mulé et al., 2009). Indeed, highlighting the relevance of LGBTQ2S+ poverty as a site of analysis and intervention, recent studies in this area have foregrounded material disparities among sexual and gender minorities in domains such as income (Waite, 2015), housing (Frederick et al., 2011; Kidd et al., 2017), and employment (Ross et al., 2016).

Despite emerging literature on the systemically poor socioeconomic conditions of LGBTQ2S+ groups, social work scholarship in this area continues to be limited. This gap in inquiry, both within social work and across the social sciences, may be attributable to the myth of “gay affluence,” or the unsubstantiated notion that gay men are affluent by virtue of having access to disposable income, which continues to prevail among scholars and is frequently generalized to other LGBTQ2S+ populations (Albelda et al., 2009; Badgett, 2003; Mulé et al., 2009). In light of the historically central role of social work in developing and promoting responses to poverty across countless social contexts as part of its commitment to social justice (Watts & Hodgson, 2019), the material inequities of LGBTQ2S+ populations require urgent consideration within the discipline. In particular, given the dearth of social work practice literature on LGBTQ2S+ poverty, there remains a need to draw on the nascent, yet growing evidence in this field of study to inform professional practice. In this article, we aim to use the findings of a recent review of the limited literature on LGBTQ2S+ poverty (Kia et al., 2020), which we completed in 2019, to conceptualize potential social work interventions addressing this issue at multiple levels of practice.

It is important to note that our review involved a synthesis of the Canadian scholarship on LGBTQ2S+ poverty specifically. However, we believe the insights yielded from this study may, for the purpose of informing social work practice, represent some transferability to the professional contexts of other Organization for Economic Co-operation and Development (OECD) member states, given comparable social infrastructures and similar human rights protections for sexual and gender minorities across many of these jurisdictions. In particular, our work may be relevant to social work practice in other states with universal health care and social programs, and those with nationally legislated protections against discrimination for LGBTQ2S+ people in domains such as employment, housing, and marriage. It is important to note that for social work practitioners operating in the U.S. context specifically, the work of DeFilippis (2016), which outlined social work calls to action in the area of LGBTQ2S+ poverty, may be useful as a guide for interventions in this jurisdiction. Given significant differences between the U.S. and Canadian context, particularly in relation to the availability of universal health care as a potential “buffer” against the impacts of LGBTQ2S+ poverty in the latter jurisdiction, this work may serve as an important complement to our article.

We begin our article by first describing our intersectional narrative review of the Canadian literature on LGBTQ2S+ poverty (Kia et al., 2020) and by presenting some of the key insights reflected in this work. Following this overview, we introduce and employ the theoretical frameworks of intersectionality (Cho et al., 2013; McCall, 2005; Yuval-Davis, 2006) and relational poverty analysis (Feldman, 2019; Piven, 2006) to conceptualize social work interventions intended to address adverse material conditions in sexual and gender minority populations. Given the social justice underpinnings of the social work profession (Watts & Hodgson, 2019), and the utility of both intersectional and relational poverty lenses for theorizing social justice work (Feldman, 2019; Watts & Hodgson, 2019), we draw on these traditions of scholarship to inform our analysis. Specifically, we use intersectionality to foreground distinct expressions of poverty affecting diverse LGBTQ2S+ groups that were represented in our review, and use relational poverty analysis to theorize responses to these manifestations of poverty in sexual and gender minority communities at various levels of social work intervention.

## LGBTQ2S+ Poverty in Canada: An Intersectional Narrative Review

In 2019, we completed an intersectional narrative review on LGBTQ2S+ poverty, which entailed the collection and synthesis of available peer-reviewed and gray literature reporting socioeconomic data on sexual and gender minorities in Canada published between 2000 and 2018. The results of this review revealed distinct material disparity in younger and older LGBTQ2S+ populations, among sexual and gender minorities identifying as racialized and/or newcomers, and among Indigenous LGBTQ2S+ people. A total of 39 works were incorporated into our analysis; a condensed list of publications comprising the basis of our analysis is included here as Table 1, and a comprehensive table of key insights reflected in each of the included works is published elsewhere (Kia et al., 2020).

**Table 1.** Summary of Works Included for Review.

Author(s)	Place	Category	Time	Sample	Methods	Poverty-Related Variables and/or Phenomena Investigated
Abichahine and Veenstra (2016)	Canada	Peer-reviewed/LGBTQ2S+ life course factors and poverty	2003–2005	*n* = 149,574 (2,526 lesbian, gay, bisexual respondents, or 1.7% of the sample)	QUAN	LGB identities, education
Abramovich (2014)	Toronto	Gray literature/poverty in LGBTQ2S+ youth	2010–2012	*n* = 34 (12 LGBTQ-identified youth [ages 16–29])	QUAL	LGBTQ youth experiences with shelter systems
Abramovich (2016)	Toronto	Peer-reviewed/poverty in LGBTQ2S+ youth	2010–2012	*n* = 32 (11 LGBTQ-identified youth [ages 16–29])	QUAL	LGBTQ2S youth experiences with shelter systems
Abramovich (2017)	Toronto	Peer-reviewed/poverty in LGBTQ2S+ youth	2010–2012	*n* = 33 (11 LGBTQ-identified youth [ages 16–29])	QUAL	LGBTQ2S youth experiences with shelter systems
Adam and Rangel (2015)	Toronto	Peer-reviewed/poverty in the migration exp. of racialized LGBTQ+ newcomers	NR	*n* = 25 Spanish-speaking men who had had sex with another man at least once in the 12 months preceding the study	QUAL	Migration experiences and social/economic capital
Adam and Rangel (2017)	Toronto	Peer-reviewed/poverty in the migration exp. of racialized LGBTQ+ newcomers	NR	*n* = 25 Spanish-speaking men who had had sex with another man at least once in the 12 months preceding the study	QUAL	Migration experiences, family/social support, social/economic capital
Alessi (2016)	Toronto	Peer-reviewed/poverty in the migration exp. of racialized LGBTQ+ newcomers	2014–2015	*n* = 26 adults who had been granted refugee, asylee, or withholding of removal status in the United States or Canada as a result of sexual orientation or gender identity; 10 participants were based in Canada	QUAL	Resettlement challenges (including those related to housing and employment) among LGBTQ+ refugees and asylees
Bergeron and Senn (2003)	Canada	Peer-reviewed/LGBTQ2S+ life course factors and poverty	NR	*n* = 254 (all lesbian-identified women)	QUAN	Lesbian identity, educational attainment, feminism, health care utilization
Brennan et al. (2013)	Ontario	Peer-reviewed/poverty in older LGBTQ2S+ adults	NR	*n* = 1,103 older adults (ages >49) living with HIV, including 726 gay men and 76 bisexual men)	QUAN	Gay and bisexual identity among men, income, educational attainment
Frederick et al. (2011)	Toronto	Peer-reviewed/poverty in LGBTQ2S+ youth	2005–2006	*n* = 147 homeless and street-involved youth (ages 16–21), with 44 (30%) identifying as nonheterosexual	QUAN	LGBTQ2S+ identities, street economy involvement, victimization measures, drug use, mental health outcomes
Gattis (2011, 2013)	Toronto	Peer-reviewed (2011)/gray literature (2013)/poverty in LGBTQ2S+ youth	2009	*n* = 147 homeless youth (ages 16–24), with 66 identifying as sexual minorities	QUAN	Sexual minority status, substance use, mental health, sexual risk behaviors, school engagement, stigma, discrimination among homeless youth
George et al. (2007)	Vancouver and Montreal	Peer-reviewed/poverty, race, and newcomer status in studies of LGBTQ+ health	1995–2003	*n* = 1,148 self-identified MSM. The Vancouver sample was limited to men aged 15–30, whereas men aged 16+ were included in the Montreal sample	QUAN	Sexual behavior, race, country of birth, income, education
Iwasaki et al. (2005)	Western Canadian city (not otherwise specified)	Peer-reviewed/poverty at the intersection of LGBTQ2S+ identity and Indigeneity	NR	*n* = 78, with 26 identifying as First Nations (17) or Métis (9)	QUAL	Lesbian/gay identities, First Nations/Métis identities, experiences with social exclusion and resilience
Jewell and Morrison (2010)	Western Canada	Peer-reviewed/LGBTQ2S+ life course factors and poverty	NR	*n* = 286 undergraduate students	MIXED	Negative attitudes and behaviors of undergraduate students toward lesbians and gay men in a mid-sized campus environment
Kidd et al. (2017)	Canada	Peer-reviewed/poverty in LGBTQ2S+ youth	2015	*n* = 1,103 homeless youth (ages 12–27), with 310 (28%) self-identifying as sexual and/or gender minorities	QUAN	Quality of life, mental illness symptoms, suicide attempt, substance use, resilience among homeless youth
Kojima (2014)	Vancouver	Peer-reviewed/poverty in the migration exp. of racialized LGBTQ+ newcomers	2009–2012	*n* = 14 gay and bisexual men, along with one trans woman, and other participants with identities representing sexual/gender diversity	QUAL	Immigration and settlement among adults representing intersections of sexual and gender diversity
Lachowsky et al. (2017)	Vancouver	Peer-reviewed/poverty, race, and newcomer status in studies of LGBTQ+ health	2012–2014	*n* = 719 gay, bisexual, and other men who have sex with men (GBM)	QUAN	GBM status, mental health, race, immigration status, income
Lessard et al. (2016)	Montreal	Peer-reviewed/poverty, race, and newcomer status in studies of LGBTQ+ health	2012–2013	*n* = 1,353 MSM	QUAN	MSM status, immigration status, income, education
Lewis (2012)	Ottawa	Peer-reviewed/LGBTQ2S+ life course factors and poverty	2010–2011	*n* = 48 self-identified gay men, with 24 located in Ottawa and another 24 in Washington, DC	QUAL	The influence of intersectional subjectivities (e.g., race, class) on migration narratives of gay men who relocated to major urban centers
Logie et al. (2016)	Toronto	Peer-reviewed/poverty in the migration exp. of racialized LGBTQ+ newcomers	NR	*n* = 29 African and Caribbean LGBTQ+ newcomers, divided into three focus groups	QUAL	Social support group participation among African and Caribbean LGBTQ+ newcomers
Lyons et al. (2016)	Vancouver	Peer-reviewed/poverty at the intersection of LGBTQ2S+ identity and Indigeneity	2012–2013	*n* = 32 trans women and two-spirit individuals, with 22 identifying as First Nations or Métis	QUAL	Trans women and two-spirit persons’ experiences with women-specific health and housing services
McCreary Centre Society (2006)	British Columbia	Gray literature/poverty at the intersection of LGBTQ2S+ identity and Indigeneity	2000	*n* = 762 street-involved youth aged 12–18, 410 of whom identified as Aboriginal	QUAN	Aboriginal identity, LGB identities, homelessness
Millett et al. (2012)	Canada (meta-analysis of Canadian, U.S., and UK literature)	Peer-reviewed/poverty, race, and newcomer status in studies of LGBTQ+ health	1981–2011	*n* = 7 studies from Canada	QUAN	MSM status, race, income
Murray (2014)	Toronto	Peer-reviewed/poverty in the migration exp. of racialized LGBTQ+ newcomers	2011–2012	*n* = 54 LGBT-identified refugee claimants, approximately 90% of whom were from Caribbean or African source countries	QUAL	Classed experiences of LGBT refugee claimants with Canada’s refugee determination system
Nakhaie and Wijesingha (2015)	Canada	Peer-reviewed/poverty, race, and newcomer status in studies of LGBTQ+ health	2004	*n* = 24,000 (respondents of Canada’s 2004 General Social Survey)	QUAN	Sexual orientation, discrimination (including workplace discrimination), health
Nierobisz et al. (2008)	Canada	Gray literature/LGBTQ2S+ life course factors and poverty	1965–2005	442 sexual orientation human rights complaints and other documentary evidence	QUAL	The role of the Canadian Human Rights Commission in advancing sexual orientation employment and other equality rights in Canada
Ploeg et al. (2013)	Hamilton	Peer-reviewed/poverty in older LGBTQ2S+ adults	2003–2005	*n* = 87 adults aged 60 and over (divided into 10 focus groups, with one focus group comprising six lesbian-identified women)	QUAL	Material disparities experienced among lesbian women in older age as reflective of structural violence/abuse
Ristock et al. (2010)	Winnipeg	Gray literature/poverty at the intersection of LGBTQ2S+ identity and Indigeneity	NR	*n* = 24 Aboriginal two-spirit, LGBTQ or MSM/WSW adults who had had experience with moving/migration	QUAL	Aboriginal two-spirit and/or LGBTQ adults’ experiences with poverty in the context of migration
Ristock et al. (2011)	Vancouver	Gray literature/poverty at the intersection of LGBTQ2S+ identity and Indigeneity	2009	*n* = 25 Aboriginal two-spirit, LGBTQ or MSM/WSW adults who had had experience with moving/migration	QUAL	Aboriginal two-spirit and/or LGBTQ adults’ experiences with poverty in the context of migration
Robinson (2017)	Two Ontario cities	Peer-reviewed/poverty at the intersection of LGBTQ2S+ identity and Indigeneity	NR	*n* = 21 Aboriginal individuals who identified as two-spirit, LGBTQ, or with other sexually/gender diverse identities	QUAL	Aboriginal two-spirit and/or LGBTQ individuals’ experiences with their social context
Ross et al. (2016)	Ontario	Peer-reviewed/LGBTQ2S+ life course factors and poverty	2012–2013	*n* = 302 bisexual adults aged 25 and over	MIXED	Bisexual identity, poverty, mental health
Saewyc et al. (2007)	British Columbia	Gray literature/poverty in LGBTQ2S+ youth	1992–2003	Approximately 90,000 secondary school students representing three cycles of the BC Adolescent Health Survey (1992, 1998, 2003 [*n* = ∼30,000 in each cycle])	QUAN	LGB identities, school experiences
Scheim et al. (2013)	Ontario	Peer-reviewed/poverty at the intersection of LGBTQ2S+ identity and Indigeneity	2009–2010	*n* = 433 trans-identified individuals across Ontario, with 32 respondents identifying as First Nations, Métis, or Inuit	QUAN	Trans identity, Aboriginal (First Nations/Métis/Inuit) identity, income
Taylor (2009)	Manitoba and Northwest Ontario	Gray literature/poverty at the intersection of LGBTQ2S+ identity and Indigeneity	2006	*n* = 75 transgender and two-spirit individuals, of whom 27 identified as Aboriginal	MIXED	Safety and security concerns, overall experiences, and service/support needs of transgender and two-spirit Aboriginal participants
Taylor et al. (2011)	Canada	Gray literature/poverty in LGBTQ2S+ youth	2007–2009	*n* = 3,607 high school students across Canada	QUAN	LGBTQ2S+ identities, school experiences
Veale et al. (2015)	Canada	Gray literature/poverty in LGBTQ2S+ youth	2013–2014	*n* = 932 trans-identified youth aged 14–25	QUAN	Trans identities, school experiences, mental health
Waite (2015)	Canada	Peer-reviewed/LGBTQ2S+ life course factors and poverty	2001–2016	Confidential 20% samples of Canada’s 2001 and 2006 census, and the 2011 National Household Survey	QUAN	Same-sex households, income
Young et al. (2012)	Canada	Peer-reviewed/LGBTQ2S+ life course factors and poverty	2009–2011	*n* = 1,552 medical students, with 81 identifying as nonheterosexual and only three identifying as non-cisgender	QUAN	Demographic characteristics of medical students, including those pertaining to sexual and gender identity

*Note*. LGBTQ2S+ = lesbian, gay, bisexual, transgender, queer, two-spirit, and other sexual and gender minority; MSM = men who have sex with men; WSW = women who have sex with women.

Our review of the literature was narrative, meaning it entailed an inductive and iterative approach to developing the central aims and scope of the study, determining eligibility criteria, and collecting and analyzing the literature (Cook, 2008; Cook & West, 2012). Narrative reviews are similar to systematic reviews in that they typically involve a methodical search, synthesis, and appraisal of available literature, but are dissimilar in that they do not incorporate aspects of deductive research design such as hypothesis testing, fixed inclusion criteria, and quantitative meta-analysis (Cook & West, 2012). Given not only the heterogeneity of populations represented in the scholarship on LGBTQ2S+ poverty, but also the range of quantitative and qualitative methodologies used, and of outcome measures and phenomena studied (e.g., educational attainment, income, employment, housing, and others), a systematic approach to this study was neither possible nor desired. The goal, initially, was to synthesize and appraise the literature on LGBTQ2S+ poverty in Canada to identify strengths and shortcomings in this body of work and to use our analysis to inform further research and identify implications for policy and practice. As we launched our search and began our collection and analysis of the literature, we noticed distinctive and intersectional expressions of poverty across the life course, and among racialized, newcomer, and Indigenous groups, and refined our aims accordingly to foreground these particular manifestations of LGBTQ2S+ poverty.

### Search Strategy

Using the support of a professional librarian, the senior author (LER) designed and led a systematic search of MEDLINE, PsycINFO, Sociological Abstracts, Social Science Abstracts, EconLit Search, and Google (for gray literature), and utilized a comprehensive list of search terms comprising keywords pertaining to LGBTQ2S+ identities, poverty, and Canada to execute the search (the list of search terms is available as Table 2 on request from the corresponding author). To be included at the initial screening stage, works needed to be written in English, to contain at least one search term from each of three keyword categories (LGBTQ2S+ identities, poverty, and Canada), and to have been published between January 1, 2000 and May 1, 2018. We did not include works predating the Year 2000, given the dearth of scholarship before then, and the reduced applicability of this research to a contemporary context of increased protections for sexual and gender minorities in domains such as employment and housing (Albert & Williams, 1998; Chuang et al., 1989). Altogether, 365 works were incorporated into the preliminary screening stage.

### Study Selection

Using Covidence (www.covidence.org), a systematic review application, we undertook a two-stage study selection process involving title/abstract and full-text screening. In the preliminary title/abstract screening stage, we looked for and selected sources that presented empirical data on socioeconomic status (e.g., income, wealth, access to benefits, education, homelessness, and housing) and/or poverty (e.g., lived experiences of poverty) among LGBTQ2S+ populations in Canada. Each source was screened for eligibility by two team members and, after differences in opinion were resolved by the senior author (LER), 102 works were selected for the second part of the study selection process.

At the full-text screening stage, we refined our inclusion criteria based on patterns we observed in the literature during title/abstract screening. Namely, given that we noticed particularly prominent and/or distinctive manifestations of material disparity in younger (ages <25; Frederick et al., 2011; Gattis, 2011), older (ages >49; Brennan et al., 2013; Ploeg et al., 2013), racialized and/or newcomer (George et al., 2007), and Indigenous (Robinson, 2017) LGBTQ2S+ populations, we included works foregrounding expressions of poverty in these groups. Of note, we included literature that presented data on the conditions and experiences of younger LGBTQ2S+ people in the school system and higher education, given known links between poor educational attainment and/or school experiences and poverty in sexual minority adults (Bergeron & Senn, 2003). This second stage of the study selection process yielded a total of 39 works for inclusion, which formed the basis of our analysis.

### Key Findings

Broadly, our review focused on conceptualizing the qualitatively distinctive expressions of material disparity among younger (ages <25; Frederick et al., 2011; Kidd et al., 2017), older (ages >49; Ploeg et al., 2013), racialized and/or newcomer (Millett et al., 2012), and Indigenous (Ristock et al., 2011; Robinson, 2017) LGBTQ2S+ people in Canada. In addition, manifestations of poverty appeared to be gendered in that they appeared to disproportionately affect women (Nakhaie & Wijesingha, 2015), along with trans and gender diverse populations (Saewyc et al., 2007; Taylor et al., 2011). Although, given the limits of narrative review methodology, we were unable to draw any global inferences about the significance of poverty at these intersections, we were able to examine patterns made apparent to us in our search, selection, and analytical processes to determine what made these intersections qualitatively rich and differentiated as sites of analysis and intervention. A summary of the findings is presented in Table 3.

**Table 3.** Summary of Findings.

Specific Subdomain	Key Insights
LGBTQ2S+ poverty throughout the life course	Sexual and gender minority youth experience systemically adverse social conditions in education systems, including discrimination and harassment, lowering the likelihood for these groups to benefit equitably from education as a mechanism of social mobility (Abichahine & Veenstra, 2016; Bergeron & Senn, 2003; Jewell & Morrison, 2010; Saewyc et al., 2007; Taylor et al., 2011; Veale et al., 2015; Young et al., 2012). LGBTQ2S+ youth experience potentially higher rates of homelessness, relative to non-LGBTQ2S+ youth, and are likely to fare worse on measures of physical and mental health, relative to their heterosexual and cisgender counterparts (Frederick et al., 2011; Gattis, 2011, 2013; Kidd et al., 2017); LGBTQ2S+ youth are also likely to experience stigma and discrimination in systems of emergency housing (Abramovich, 2014, 2016, 2017). Older LGBTQ2S+ adults, particularly women, may be affected by lifetime exposure to inequities in income and employment while simultaneously fearing vulnerability to violence, stigma, and discrimination if poor living conditions force them to transition into contexts of institutional care (Brennan et al., 2013; Ploeg et al., 2013). Socioeconomic penalties appear to be incurred among bisexuals who conceal their identities, whereas lesbians/gay men may instead experience such penalties upon coming out, particularly in early adulthood (Ross et al., 2016; Waite, 2015).
LGBTQ+ poverty, race, and newcomer status	Racialized LGBTQ+ communities in Canada experience income and other socioeconomic disparities relative to their white counterparts (George et al., 2007; Lachowsky et al., 2017; Lessard et al., 2016; Millett et al., 2012; Nakhaie & Wijesingha, 2015). Some racialized newcomer LGBTQ+ people may be uniquely likely to tolerate poor socioeconomic conditions in Canada, including precarious employment, particularly given fears of experiencing persecution based on LGBTQ+ identity if they return to their countries of origin (Adam & Rangel, 2015, 2017; Alessi, 2016; Kojima, 2014). Organizations mandated to support LGBTQ+ newcomers function both as sources of support (Logie et al., 2016) and as structures involved in the exploitation of those in this group (Murray, 2014).
LGBTQ2S+ poverty and Indigeneity	Indigenous LGBTQ2S+ persons experience profound material disparities relative to other LGBTQ+ groups in domains such as housing and employment (McCreary Centre Society, 2006; Ross et al., 2016; Scheim et al., 2017; Taylor, 2009). Cultural groups may function as sources of strength and resilience against poverty for Indigenous LGBTQ2S+ people (Iwasaki et al., 2005; Robinson, 2017). Indigenous LGBTQ2S+ peoples’ experiences of homo/bi/transphobia in Indigenous contexts, and colonial racism in LGBTQ+ communities and Canadian society at large, isolate these groups from the formal and informal systems of support often required to mitigate poverty (Lyons et al., 2016; Ristock et al., 2010, 2011; Robinson, 2017).

*Note*. LGBTQ2S+ = lesbian, gay, bisexual, transgender, queer, two-spirit, and other sexual and gender minority.

In studies of youth populations under the age of 25, sexual and gender minorities reported prominent experiences of stigma and discrimination in school systems, which in turn negatively impacted their ability to utilize education as a mechanism of social mobility (Saewyc et al., 2007; Taylor et al., 2011). Illustrating gendered differences in these data, Saewyc et al. (2007) found that lesbian and bisexual girls reported feeling less connected to their school than their heterosexual counterparts, and that although gay and bisexual boys similarly felt connected to their school less than their heterosexual male peers, disparities in connectedness were significantly greater for sexual minority girls than for sexual minority boys. Aside from the findings related to younger LGBTQ2S+ people’s experiences with education, our review of this literature revealed that younger sexual and gender minorities experience potentially higher rates of homelessness than their non-LGBTQ2S+ counterparts (Kidd et al., 2017), and that LGBTQ2S+ youth experiencing homelessness may fare worse on measures of physical and mental health relative to their heterosexual and cisgender peers (Frederick et al., 2011; Kidd et al., 2017).

At the other end of the life course, although we found minimal scholarship addressing the material conditions of older LGBTQ2S+ adults, the small body of literature in this area (Brennan et al., 2013; Ploeg et al., 2013) foregrounded distinct manifestations of poverty among those in this population. For example, in one qualitative study, older lesbians discussed their experiences with adverse socioeconomic conditions, including lifetime gendered disparities in access to adequate employment and income, as well as the contemporary homophobic conditions in residential care facilities intended for older adults, as prominent factors shaping their distinctive susceptibility to elder abuse (Ploeg et al., 2013). With respect to other life course factors, one interesting finding was that although lesbians and gay men appeared to report penalties in income after “coming out” (i.e., disclosing their sexual identities), typically in early adulthood (Waite, 2015), comparable penalties in income appeared to be incurred among bisexual adults who concealed their identities (Ross et al., 2016).

In our review of the literature reporting socioeconomic data on LGBTQ+ newcomers and racialized communities, we found a number of unique manifestations of poverty reflected in the social conditions and experiences of those in these groups. First, particularly in studies of sexual health among men who have sex with men (MSM), we found that racialized MSM frequently report disparities in income and employment relative to their white counterparts (George et al., 2007; Lessard et al., 2016; Millett et al., 2012). For example, in one meta-analysis of the health behaviors and health outcomes of MSM across the United States, Canada, and the United Kingdom, Canadian Black MSM appeared to be approximately 1.56 times more likely than their non-Black counterparts to report incomes under US$20,000 (Millett et al., 2012).

In much of the qualitative literature addressing socioeconomic issues in racialized newcomer LGBTQ+ populations, we found that sexual and gender minorities in these groups commonly reported tolerating precarious economic conditions in Canada, including unemployment and underemployment, to avoid having to return to source countries with potentially hostile conditions for LGBTQ+ groups (Adam & Rangel, 2015, 2017; Alessi, 2016; Kojima, 2014). Interestingly, one qualitative study indicated that systems of settlement support intended for LGBTQ+ newcomers appeared to assist individuals in this group broker resources such as housing and employment and thus mitigate conditions of poverty (Logie et al., 2016). Another study, however, illustrated the tendency for some typically white-led LGBTQ+ community organizations to exploit the labor of sexual and gender minority refugees by asking them to perform free/voluntary work in exchange for granting them reference letters frequently required by the Canadian refugee determination system to validate the claims of sexual and gender minorities fleeing persecution (Murray, 2014). Although not all racialized people are newcomers, and not all newcomers are racialized, the literature regularly reported data on these two groups simultaneously, which in turn required us to consider and analyze the poverty-related issues of these two groups together.

In appraising the small body of literature reporting socioeconomic data on Indigenous LGBTQ2S+ populations in Canada, we found that expressions of poverty in this context were salient across numerous domains and shaped by intersecting systems of oppression uniquely targeting those in these groups. For example, the scholarship in this area foregrounded the tendency for Indigenous sexual and gender minorities to report significantly poorer outcomes in income (Ross et al., 2016; Taylor, 2009), education (Taylor, 2009), and housing (Lyons et al., 2016) relative to their non-Indigenous counterparts, and commonly attributed such disparities to the potential intersectional effects of settler colonialism, racism, and homo/bi/transphobia on the socioeconomic conditions of these groups (Lyons et al., 2016; Ross et al., 2016).

Some of these studies highlighted that many Indigenous sexual and gender minority people experience stigma and discrimination against their LGBTQ2S+ identities in Indigenous contexts while also encountering settler colonialism and racism in mainstream LGBTQ2S+ settings (Ristock et al., 2010, 2011; Robinson, 2017). Given the significant role of informal social support in enabling individuals and communities to overcome socioeconomic hardship (Robinson, 2017; Ross et al., 2016), this literature highlighted low social support among Indigenous sexual and gender minorities as an important impediment for social mobility and poverty alleviation in this context (Ristock et al., 2010, 2011; Robinson, 2017). A source of strength and resilience reflected in the literature on poverty among Indigenous LGBTQ2S+ people was the critical function of belonging to Indigenous cultural and community groups for mitigating social exclusion as a possible determinant of poverty (Iwasaki et al., 2005).

## Intersectionality and Relational Poverty as Theoretical Frameworks

We draw on intersectionality (Bowleg, 2008; Cho et al., 2013; McCall, 2005; Yuval-Davis, 2006) and relational poverty (Feldman, 2019; Lawson & Elwood, 2014; Piven, 2006) as key theoretical frameworks to inform the analysis we present in this article. While we utilize the former to conceptualize the distinctive manifestations of poverty among diverse sexual and gender minorities represented in this review, we use the analytical lens of relational poverty to develop social work interventions that seek to address material disparities at various levels of practice. Given that both intersectionality and relational poverty enable scholars to center, interrogate, and take up material and immaterial (e.g., discursive) marginalization as sites of social change (Feldman, 2019; Yuval-Davis, 2006), these lenses are compatible with our goal of informing social work responses to LGBTQ2S+ poverty that are driven by an overarching commitment to social justice. We would like to note that post hoc application of theoretical frameworks, specifically to conceptualize the findings of narrative review studies, is commonplace, given the iterative and inductive design of such inquiry (Baumeister & Leary, 1997).

Intersectionality, as a theoretical framework, originates in the oral and written activism and thought of Black (Collins, 2009; Combahee River Collective, 1977; Crenshaw, 1989) and Indigenous (Clark, 2016) women in North America, who have problematized “single axis” models of oppression that render invisible the distinctive conditions of marginalization on multiple grounds (e.g., race and gender). These leaders have emphasized the importance of moving beyond “single axis” analyses to understand the social conditions and experiences of multiply marginalized groups as sources of knowledge construction and sites of social change (Collins, 2009; Yuval-Davis, 2006). Although much intersectional thought is centered on examining the interplay of race- and gender-based oppression in the lives of racialized women, this framework is also used to investigate the issues and experiences of other multiply marginalized groups, including those of sexual and gender minorities (Bowleg, 2008; Clark, 2016; Cronin & King, 2010).

McCall (2005) has categorized much of the contemporary intersectional scholarship as falling into three types of analysis: intercategorical, intracategorical, and anticategorical. While intersectional inquiry that takes up intercategorical complexity largely focuses on social, health, and other disparities between multiply marginalized groups and dominant reference populations, intracategorical scholarship is concerned with identifying sources of heterogeneity *within* a group affected by intersecting systems of oppressions. Anticategorical analysis, lastly, is centered on identifying and deconstructing essentialist and homogenizing categories of identity to which multiply marginalized groups are often relegated. In our work, we consider both intercategorical and anticategorical complexity in that we highlight salient material disparities between sexual and gender minorities and non-LGBTQ2S+ reference populations and also challenge homogenizing notions of “LGBTQ2S+” as a monolithic identity by presenting differences in expressions of poverty across groups represented by this acronym. However, our work is primarily intracategorical as it seeks, foremost, to foreground distinctions in conditions and experiences of LGBTQ2S+ poverty along dimensions such as age, race, and Indigeneity as sites of social work intervention and social justice work.

Relational poverty, a second framework on which we draw to inform our analysis, focuses on social, economic, and political relationships between those in poverty and those in relative positions of power and influence (Elwood et al., 2017; Feldman, 2019). This theoretical tradition is compatible with intersectionality in that it recognizes the role of intersecting power relations pertaining to class, race, and gender, along with other dimensions of difference, in creating and sustaining conditions of poverty for marginalized people, and identifies these relationships as targets of social action (Elwood et al., 2017; Garrett, 2017). For example, scholars engaging in relational poverty analysis sometimes analyze how the material dispossession of land, among some marginalized peoples, intersects with racialization, gendering, and other social processes to reproduce or deepen conditions of disparity for these groups (Elwood et al., 2017). Instead of focusing on the material conditions of impoverished groups exclusively, relational poverty analysis necessitates attention to actors involved in creating and maintaining material disparities, including middle class professionals, policy makers, and the wealthy entrepreneurial elite, as potential targets of intervention (Piven, 2006).

Although scholarship incorporating relational poverty analysis often foregrounds material disparities between the poor and nonpoor (Lawson & Elwood, 2014; Piven, 2006), the role of discursive factors in generating and normalizing poverty is also taken up as an area of inquiry and action (Feldman, 2019; Garrett, 2017). For example, Garrett’s (2017) work has drawn attention to dominant discourses that construct racialized single mothers receiving publicly administered financial assistance as morally and normatively transgressive actors whose “dependence” on the welfare state requires surveillance and discipline. This scholar has, through this work, theorized the importance of social and political activities that draw on the resistance of people living in poverty to challenge hegemonic and often punitive discourses, as well as those that catalyze and promote the alleviation of material inequities.

In the rest of this article, we employ an intersectional lens to foreground and conceptualize distinctive expressions of poverty in LGBTQ2S+ populations in our literature review as sites of social work intervention. Following this, we draw on relational poverty, using insights developed in the preceding section, to consider social work interventions addressing poverty at various levels of practice, ranging from “micro” to “macro” . Given the focus of relational poverty on addressing power relations that create and reinforce conditions of material inequity, our social work interventions center social workers as potentially influential middle-class actors with capacity to promote change in LGBTQ2S+ poverty, in solidarity with sexual and gender minorities affected by these conditions.

## LGBTQ2S+ Poverty as a Site of Social Work Intervention

Although the Canadian literature on LGBTQ2S+ poverty is limited, it foregrounds material inequities at several intersections across the life course, race and newcomer status, and Indigeneity. Drawing on an intersectional lens (Cho et al., 2013; McCall, 2005; Yuval-Davis, 2006), and incorporating intracategorical analysis in particular (McCall, 2005), several issues merit attention as sites of social work intervention. As we believe social justice to be a core tenet of social work’s historical response to poverty (Watts & Hodgson, 2019), and since adoption of an intersectional lens is well-suited to informing social work practices shaped by a commitment to social justice (Mattsson, 2014), we define “social work intervention” as a form of social justice work.

What is most apparent to us in this literature review is the scarcity of scholarship addressing LGBTQ2S+ poverty (Kia et al., 2020). Although the results of this review are limited to the Canadian context, scholars reporting on poverty among sexual and gender minorities in other jurisdictions (Albelda et al., 2009) report a comparable lack of inquiry. Given this, we foreground the invisibility of LGBTQ2S+ poverty as an issue requiring urgent attention in social work practice. Utilizing an intracategorical lens (McCall, 2005), and drawing on conceptual literature addressing material inequities among sexual and gender minorities (Albelda et al., 2009; Mulé et al., 2009), we see this absence of scholarship on LGBTQ2S+ poverty as being reflective of the reach and impact of “gay affluence” as a dominant discourse erroneously generalized to LGBTQ2S+ populations, which in turn obscures socioeconomic disparities in sexual and gender minorities other than middle-aged white and cisgender gay men.

Emphasizing the intracategorical differences homogenized under the “LGBTQ2S+” umbrella, we note the dangers of the myth of “gay affluence” and draw attention to domains silenced under this discourse as areas for urgent intervention. We emphasize the need to recognize socioeconomic disparities among younger and older sexual and gender minorities, along with LGBTQ2S+ people who are racialized and/or newcomers, and LGBTQ2S+ people who are Indigenous (Kia et al., 2020), and argue that ignoring these issues within the social work profession reinforces these conditions of oppression.

Extending the insights above, and incorporating intracategorical analysis, we identify the need, within social work practice, to address intersecting and systemic conditions underlying these material disparities. In considering younger and older sexual and gender minorities affected by poverty, for example, we argue that it is important to acknowledge and address the intersecting role of ageism in rendering these groups susceptible to insecurities in housing (Kidd et al., 2017; Ploeg et al., 2013), along with disparities in access to education (Saewyc et al., 2007; Taylor et al., 2011). Similarly, it is critical to attend to the gendered nature of disparities at both ends of the life course, given that older lesbians, for instance, experience lifetime disparities in income and employment that result in heightened vulnerability to housing insecurity later in life (Ploeg et al., 2013), and that sexual minority girls may experience more significant inequities in accessing education than gay and bisexual boys (Saewyc et al., 2007). Finally, recognizing that gay men and lesbians who “come out” may experience socioeconomic penalties after doing so in early adulthood (Waite, 2015), while bisexuals may instead incur such penalties as a consequence of concealing their identities (Ross et al., 2016), we argue for the need to consider how life course factors differentially affect the socioeconomic conditions of sexual and gender minorities often homogenized under the “LGBTQ2S+” umbrella.

Recognizing the unique material disparities reported among racialized and newcomer LGBTQ+ people represented in our review, we note that racism intertwines with other systems of oppression affecting sexual and gender minorities such as homophobia, biphobia, and transphobia, shaping distinctive material inequities at this intersection (Adam & Rangel, 2017; Murray, 2014). We draw attention to income disparities reported among Black MSM, relative to their white counterparts (Alessi, 2016), as one effect of these interlocking social forces. Community-based social services play a dual role in supporting racialized and newcomer LGBTQ+ groups to broker resources to mitigate poverty, including access to housing and employment (Logie et al., 2016), and in potentially exploiting the labor of racialized asylum seekers who rely on these organizations to help validate their refugee claims (Murray, 2014). Given this, we recognize poverty among racialized and newcomer LGBTQ+ groups as a salient domain for constructing social work interventions more firmly grounded in a commitment to social justice.

Finally, given the adverse material conditions of Indigenous LGBTQ2S+ groups in our review, it is critical to account for the significant role of settler colonialism and racism, together with systemic stigma and discrimination targeting sexual and gender minorities, at this intersection. Settler colonialism, a term often used to describe the attempted symbolic and legislated destruction and replacement of Indigenous societies by settler invaders (Wolfe, 2006), is recognized for its impact on the social conditions of Indigenous sexual and gender minorities (Clark, 2016; Robinson, 2017). Two-spirit identities, which are culturally and historically rooted nonheterosexual and/or non-cisgender identities used by some Indigenous peoples in Canada (Lyons et al., 2016; Robinson, 2017), were denigrated at the inception of settler society and continue to be marginalized by systems of colonial power (Clark, 2016).

Engaging with this social and historical context is required, within social work practice, to understand why Indigenous LGBTQ2S+ people may be marginalized on the basis of their Indigeneity (including their two-spirit identities) across non-Indigenous LGBTQ+ settings and simultaneously subjugated as sexual and gender minorities in Indigenous communities influenced by settler colonialism (Ristock et al., 2010, 2011; Robinson, 2017). This intersectional context may help explain the low social support Indigenous sexual and gender minority people often report in Indigenous and LGBTQ2S+ settings (Robinson, 2017; Ross et al., 2016) as an impediment to overcoming conditions of poverty (Robinson, 2017). It may, lastly, offer insight on why belonging to cultural groups may be critical for poverty alleviation among Indigenous sexual and gender minorities, who often require such connection to mitigate the complex influence of settler colonialism, racism, and LGBTQ2S+ stigma/discrimination (Iwasaki et al., 2005).

A theoretically informed appreciation of the systemic forces underpinning poverty among diverse groups of LGBTQ2S+ people is necessary for conceptualizing social work interventions that mitigate these inequities while also addressing systems of oppression that create and sustain these conditions. Our use of intracategorical intersectionality (McCall, 2005) as an analytical lens provides a foundation for constructing social work interventions that curtail poverty as a material “symptom” of multiple marginalization and take up the root causes of poverty in the pursuit of social justice.

## Conceptualizing Social Work Interventions to Address LGBTQ2S+ Poverty

In this section, we conceptualize social work interventions that may challenge LGBTQ2S+ poverty at multiple levels. We begin by outlining potential interventions at the “micro” level of direct practice, followed by those at the organizational or “meso” level, and conclude with the systemic or “macro” level of social work practice. Consistent with relational poverty analysis (Feldman, 2019; Piven, 2006), the analytical framework we use to guide this part of our article, we foreground practices to change relations of power underlying the material inequities most prominently represented in our analysis of LGBTQ2S+ poverty. Although we differentiate between practices at the “micro,” “meso,” and “macro” levels of practice, we caution readers to understand that this classification of social work intervention does not, on its own, represent our application of relational poverty analysis. Instead, within each level of practice, we conceptualize ways that social workers can transform relations of power that undergird the socioeconomic disparities of sexual and gender minority populations.

### Social Work Interventions at the Level of Direct Practice

Most importantly, social workers who operate at the level of direct practice may need to consider interventions that challenge the myth of “gay affluence,” which frequently renders the poverty-related issues and needs of LGBTQ2S+ people invisible and unaddressed. Challenging “gay affluence” entails questioning discursive practices that stem from this notion, including the frequent and erroneous generalization of this myth to youth and older adults, along with those who are racialized and newcomers, and those who are Indigenous (Kia et al., 2020). It also means critically interrogating the homogenization of “LGBTQ2S+” groups, given the prominence of material disparities affecting some of the groups situated under this acronym, including trans and bisexual populations (Kia et al., 2020). In working with individual sexual and gender minority clients, particularly those belonging to the aforementioned groups, reflexivity may help ensure that social workers do not adopt this myth and change their approach if they recognize its influence in their work. For example, in health care and social service settings, social workers are frequently asked to complete psychosocial assessments and, in this process, may be tempted to overlook questions related to housing, employment, education, and income with sexual and gender minority clients, especially if they are unfamiliar with the systemic material inequities to which their client may be exposed. To use another example, social workers who primarily engage in anti-poverty work may assume that all of their clients are heterosexual and/or cisgender and thus overlook questions related to sexual orientation and gender identity. In these situations, social workers can become aware of stereotypes informing their understanding of the material conditions of LGBTQ2S+ clients and incorporate questions about economic security in their assessments. Using the lens of relational poverty (Feldman, 2019), this approach may destabilize relational norms, which are often rooted in the myth of “gay affluence,” and which normalize the invisibility of LGBTQ2S+ poverty in health care and social services (Mulé et al., 2009).

Another direct practice intervention for social workers involves advocacy for housing, employment, education, and income resources while providing seemingly unrelated “clinical” support to LGBTQ2S+ clients. Given social work’s common incorporation of the person-in-environment perspective to inform practice (Kondrat, 2002), this intervention may appear self-explanatory, yet the importance of offering practical assistance may not be as apparent to non-generalist clinicians offering specialized services such as psychotherapy or clinical case management in tertiary mental health settings. The disproportionately poor mental health outcomes of LGBTQ2S+ people, together with corresponding inequities in housing, employment, and education, have been attributed in part to the systemic exposure of these groups to stigma and discrimination (Bauer & Scheim, 2015; Millett et al., 2012; Ross et al., 2016). Clinical social workers, whose services may address the mental health impacts of stigma and discrimination as determinants of LGBTQ2S+ poverty, may find themselves uniquely positioned to assist with poverty alleviation in their work with sexual and gender minority clients. This may be the case particularly with LGBTQ2S+ individuals who experience multiple forms of subjugation based on LGBTQ2S+ identity, age (Kidd et al., 2017), race (Millett et al., 2012), newcomer status (Adam & Rangel, 2017), or Indigeneity (Ristock et al., 2010, 2011). Utilizing a relational poverty analysis lens (Feldman, 2019; Lawson & Elwood, 2014), social workers providing mental health services to LGBTQ2S+ clients, by using their power as professional middle-class actors who access and broker resources required to mitigate poverty, may challenge relations of power that render invisible, and thus reinforce, the material adversities of sexual and gender minority clients in formal systems of care. By ensuring, in particular, that the housing, employment, and other resources they broker are safe and appropriately responsive to the issues and needs of sexual and gender minority clients, social work clinicians can enhance the effectiveness and structural impact of these interventions.

### Social Work Interventions at the Level of Organizations

Practitioners primarily operating at the organizational level may shape policies and procedures that inform programming and direct practice at agencies involved in the provision of health care and social services, including policies that guide the brokerage of resources to achieve economic security, such as housing and education. Given the significance of the myth of “gay affluence” in rendering LGBTQ2S+ poverty invisible, social workers should incorporate practices that challenge this discourse and its impact on programming, policies, and procedures.

One example of the likely discursive impact of “gay affluence” on service provision is reflected in the programming of organizations that offer general emergency housing options but do not designate specialized programs for LGBTQ2S+ youth, despite the disproportionate number of homeless sexual and gender minority youth and the susceptibility of these service users to violence, stigma, and discrimination in non-LGBTQ2S+ settings (Abramovich, 2016; Kidd et al., 2017). Practitioners who encounter organizational climates such as these, where certain manifestations of LGBTQ2S+ poverty go largely unrecognized and unaddressed, may advocate for policy change to prioritize LGBTQ2S+ clients, along with tailored policies, procedures, and practices designed to meet their needs. Using the example of homeless sexual and gender minority youth, social workers employed by emergency housing providers may champion policies prohibiting discrimination against LGBTQ2S+ youth in agency settings and, in the longer term, help develop temporary housing programs for young LGBTQ2S+ clients. Applying the lens of relational poverty analysis (Feldman, 2019), a reprioritization of LGBTQ2S+ youth in programming would undermine hegemonies of invisibility that reinforce the material marginalization of younger sexual and gender minorities at the institutional level and create spaces that enable youth to resist material inequities by engaging tailored resources to mitigate poverty.

Another social work intervention situated at the “meso” level of practice is partnership development among stakeholders to address the needs of LGBTQ2S+ populations affected by multiple systems of oppressions. This intervention is especially relevant for racialized and newcomer LGBTQ+ populations, and for Indigenous LGBTQ2S+ groups. The exposure of these groups to multiple forms of stigma and discrimination, and to profound poverty related to such interlocking marginalization, substantiates collaborative responses from organizations serving LGBTQ2S+ communities, those serving racialized people and newcomers, and those serving Indigenous communities (Murray, 2014; Ristock et al., 2011). Indigenous LGBTQ2S+ people, frequently marginalized in non-Indigenous LGBTQ+ organizations and in their respective Indigenous communities (Robinson, 2017), would benefit from cross-organizational partnerships between providers and programs in particular. Given, specifically, the historical complicity of the social work profession in the colonization of Indigenous peoples and communities through institutions such as the child welfare system (Blackstock, 2019), and especially the ongoing participation of the social work profession in the colonization of Indigenous LGBTQ2S+ people through the displacement of two-spirit children, parents, and other kin on the basis of poverty, such grassroots collaboration is critical. Services that are formed from partnership development between LGBTQ2S+ organizations and Indigenous communities may promise to build foundations of community belonging and support that not only mitigate the structural conditions underpinning poverty among Indigenous sexual and gender minorities, but more importantly, also contribute to the decolonization of the social work profession. Social work practitioners should practice critical reflexivity in striking a balance between supporting such partnership development initiatives and simultaneously ensuring that these activities be led by LGBTQ2S+ and Indigenous groups, particularly if they do not have lived experience of this intersection.

From the analytical position of relational poverty (Feldman, 2019), community members should be engaged directly for guidance in the development of such partnerships, and those with lived experience in these communities should be compensated to lead and deliver relevant programming. Given that social work practice informed by relational poverty (Feldman, 2019) incorporates tangible relational interventions to shift the power dynamics underpinning poverty, engaging with and hiring community members from multiply marginalized LGBTQ2S+ groups to lead programming may be a critical step in intersectional anti-poverty work involving cross-organizational partnership development.

### Social Work Interventions at the Systemic Level

Social workers practicing at the “macro” level of policies, systems, and larger bureaucracies play a critical role in advocating for and catalyzing transformational responses to LGBTQ2S+ poverty. Given the continued dearth of scholarship on material inequities affecting sexual and gender minorities (Kia et al., 2020), social workers at this level of intervention may engage in research activities that promote insight into the breadth and depth of LGBTQ2S+ poverty. Areas warranting further attention include the prominence of LGBTQ2S+ poverty at both the beginning and later stages of the life course (Kidd et al., 2017; Ploeg et al., 2013), LGBTQ2S+ poverty among racialized and newcomer populations (Adam & Rangel, 2017), and poverty among Indigenous LGBTQ2S+ groups (Robinson, 2017). Bisexual (Ross et al., 2016) and trans individuals (Bauer & Scheim, 2015) within these groups, along with older adults (Ploeg et al., 2013), appear to be especially underrepresented in the scholarship despite being disproportionately subject to conditions of poverty. If not conducting research themselves, social workers at the “macro” level may partner with social work researchers to inform the growth of scholarship on LGBTQ2S+ poverty and should ensure that sexual and gender minorities with lived experience of poverty are centered and meaningfully engaged in informing and guiding such research. Social workers can review examples of community-based critical scholarship on HIV/AIDS (Harris, 2007) and disability rights (Yeo & Moore, 2003) to imagine how communities may be mobilized to inform research with LGBTQ2S+ groups affected by poverty. Drawing on the lens of relational poverty analysis (Feldman, 2019), more research would demonstrate the material inequities of sexual and gender minorities as expressions of social injustice and “justify” policy and practice interventions intended to destabilize, or at least address, the relational systems of oppression underpinning them.

In addition to engagement in research, social workers practicing at the “macro” level should engage with LGBTQ2S+ individuals, groups, and communities affected by poverty and amplify their voices in advocating for systems-level change. In Canada (Government of Canada, 2018), as in other OECD nation states, government agencies mandated to develop and execute anti-poverty policy frequently carry out community consultations in which stakeholders are invited to help shape, provide feedback, and develop policy in this area. Social workers at the “macro” level may seize such opportunities to build relationships and partner with LGBTQ2S+ community stakeholders, in order to encourage their participation in these processes and ensure their distinctive poverty-related issues and needs be known to policy makers. Through their professional organizations, social workers may partner with LGBTQ2S+ communities affected by poverty to develop position papers, editorials, open letters, and other advocacy tools, mobilizing their collective social capital, to draw attention to LGBTQ2S+ poverty as a site of population-level policy intervention. This approach to advocacy may be a part of a formal consultation or occur when other comparable opportunities arise. Engagement with LGBTQ2S+ communities in advocacy is critical to center the voices of marginalized sexual and gender minorities as legitimate sources of knowledge construction and social action.

## Final Remarks

Our aim in this article was to draw on the findings of a review of the Canadian literature on LGBTQ2S+ poverty to generate social work interventions that may address this issue at multiple levels of practice. We substantiated LGBTQ2S+ poverty as a site of social work practice and conceptualized interventions in this area.

It is important to emphasize the most salient limitation of our work, namely its focus on LGBTQ2S+ poverty in Canada, and thus its lack of transferability to a majority of non-OECD jurisdictions. We hope that social work practitioners and researchers with interest in addressing LGBTQ2S+ poverty can draw on this limitation to substantiate international studies of socioeconomic disparity among sexual and gender minorities and, in so doing, help develop a robust evidence base from which to refine our preliminary conceptualization of social work interventions. Given our findings regarding the social conditions and experiences of younger and older LGBTQ2S+ people, as well as racialized, newcomer, and Indigenous sexual and gender minorities, we believe practitioners and scholars outside of Canada may consider these lines of inquiry as tentative guides for exploring questions related to poverty in their respective LGBTQ+ and Indigenous sexual and gender minority communities and modify them depending on the unique conditions of their local geopolitical context.

Centering LGBTQ2S+ poverty as a site of both ongoing knowledge construction and critical policy and practice responses tailored to transform the underlying social conditions giving rise to this area of inequity is critical for ensuring social work’s approach to poverty is comprehensive and inclusive. We encourage practitioners to adopt a critically reflexive approach to LGBTQ2S+ poverty that challenges myths of “gay affluence” historically hindering work in this area. We recommend developing partnerships with sexual and gender minority communities to address material inequities affecting LGBTQ2S+ people. Working at multiple levels of intervention, using intersectional and other critical frameworks to guide practice, social workers can more fully enact the social justice underpinnings of the profession and affirm the centrality of anti-poverty work, both in LGBTQ2S+ communities and beyond, to the pursuit of social justice.
